# Supplementary material for: Maximum knee extension velocity without external load is a stronger determinant of gait function than quadriceps strength in the early postoperative period following total knee arthroplasty
Source: PLoS One. 2022 Nov 22;17(11):e0276219. doi: 10.1371/journal.pone.0276219 (PMC9681062; doi:10.1371/journal.pone.0276219)
Supplement: Attachment — Submitted filename: Revised_Response to Reviewers@2022.6.20.docx [file pone.0276219.s002.docx]

**Responses to Reviewer #1:**

We wish to express our appreciation to the reviewer for his/her helpful and insightful comments regarding our manuscript, which improved our work. According to reviewer’s suggestions, we have revised the manuscript accordingly. **(Excerpts from the reviewer’s comments are indicated in bold.)**

**Reviewer #1:** **This is an interesting study about TKR and Knee extension velocity. The study is well written, with for me, only some remarks.**

Response: We thank the reviewer for these positive comments.

***Introduction**

1. **Please add references for these two sentences: “However, in clinical situations, there are some patients whose gait function recovers to normal levels despite a considerable reduction in quadriceps strength. Conversely, some patients may have recovered to sufficient muscle strength but still have a decline in gait function. »**

Response: We appreciate the reviewer’s comment on this point. We have described these sentences based on our clinical experience. Then, we cannot add references to these. Thus, we have deleted this part and changed it as follows.

From: However, in clinical situations, there are some patients whose gait function recovers to normal levels despite a considerable reduction in quadriceps strength. Conversely, some patients may have recovered to sufficient muscle strength but still have a decline in gait function. Furthermore, some studies reported a large gap between the reduction rate in gait function and the decrease rate in quadriceps strength following TKA [4,10,18].

To: However, some studies reported a large gap between the reduction rate in gait function and the decreased rate in quadriceps strength following TKA [4,10,18]. (Lines 73-74)

1. **Please Delete this sentence : « (muscle power = muscle strength × movement velocity) »**

Response: As suggested, we deleted this sentence.

1. **Please add reference for this sentence : « Movement velocity is the speed at which the upper/lower limbs or trunk are moved as quickly as possible under various loading conditions.** »

Response: Thank you for your suggestion. We have added the references as follows.

To: Movement velocity is the speed at which the upper [21] /lower limbs [22-24] or trunk [25,26] are moved as quickly as possible under various loading conditions. (Lines 84-86)

21. Iwata A, Higuchi Y, Sano Y, Ogaya S, Kataoka M, Yonetsu R, et al. Maximum movement velocity of the upper limbs reflects maximum gait speed in community-dwelling adults aged older than 60 years. Geriatr Gerontol Int 2014;14(4): 886–889. doi: 10.1111/ggi.12186.
22. Sayers SP, Guralnik JM, Thombs LA, Fielding RA. Effect of leg muscle contraction velocity on functional performance in older men and women. J Am Geriatr Soc. 2005;53(3): 467-471. doi: 10.1111/j.1532-5415.2005.53166.x.
23. Van Roie E, Verschueren SM, Boonen S, Bogaerts A, Kennis E, Coudyzer W, et al. Force-velocity characteristics of the knee extensors: an indication of the risk for physical frailty in elderly women. Arch Phys Med Rehabil. 2011;92(11): 1827-1832. doi: 10.1016/j.apmr.2011.05.022.
24. Arai T, Obuchi S, Shiba Y, Omuro K, Inaba Y, Kojima M. The validity of an assessment of maximum angular velocity of knee extension (KE) using a gyroscope. Arch Geronto Geriatr. 2012;54(2) :e175–e180. doi: 10.1016/j.archger.2011.10.012.
25. 25.	Iwata A, Higuchi Y, Kimura D, Okamoto K, Arai S, Iwata H, et al. Quick lateral movements of the trunk in a seated position reflect mobility and activities of daily living (ADL) function in frail elderly individuals. Arch Geronto Geriatr. 2013; 56(3):482–486. doi: 10.1016/j.archger.2012.12.002.
26. Iwata A, Higuchi Y, Sano Y, Ogaya S, Kataoka M, Okuda, K, et al. Quickness of trunk movements in a seated position, regardless of the direction, is more important to determine the mobility in the elderly than the range of the trunk movement. Arch Geronto Geriatr. 2014;59(1): 107–112. doi: 10.1016/j.archger.2014.02.001.

***Methods
Measurements:**

1. **I think that it could be important to have a description of the type of implant used and surgery approach used also and the number of surgeons because there is a real impact on TKA, recovery, quadriceps strength …. We know nothing about this.**

**In addition, it is also important to explain the rehabilitation session received by the patient after TKA. As surgery technique and type of implant, there is a large impact of this rehabilitation on recovery especially after such a short time after TKA.**

Response: Thank you for pointing this out. We have added a description about the number of surgeons, surgical approach, type of implants, and rehabilitation program at the beginning of the Results section as follows.

To:

A total of 186 patients participated in this study. The operation was performed by 14 surgeons at four hospitals, using a medial parapatellar approach in 182 cases and mini-midvastus approach in four cases. The type of implant utilized was posterior-stabilized in 121 cases, rotational platform in 30 cases, posterior cruciate-retaining in 17 cases, posterior cruciate-substituting in two cases, and unknown in 16 cases. All patients underwent a standard rehabilitation program per institutional protocol with a physical therapist 5 days a week for 3 weeks the day after TKA. (Lines 184-190)

1. **Add BMI in your list. Maybe it is sufficient to have only BMI and height and weight--**

Response: This has been revised as suggested by the reviewer.

From: We collected demographic information, including age, sex, height, and weight, for each participant.

To: We collected demographic information, including age, sex, height, weight, and body mass index (BMI) for each participant. (Lines 111-112)

1. **Please define TUG and add references to justify that it is a good test to evaluate gait function.**

Response: As suggested by the reviewer, we added a sentence as follows.

To: TUG is a reliable and valid test to quantify functional mobility in older adults [28]. (Lines 114-115)

28. Podsiadlo D, Richardson S. The timed "Up & Go": a test of basic functional mobility for frail elderly persons. J Am Geriatr Soc. 1991;39(2): 142-148. doi: 10.1111/j.1532-5415.1991.tb01616.x.
2. **Please add for ROM, that it is a PASSIVE ROM….. indeed, we can think of the active measurement made during walking.**

Response: As the reviewer noted, our original expression was confusing. We clarified that it was a passive ROM.

From: We measured the knee extension velocity, quadriceps strength, range of motion (ROM), and pain to evaluate knee function.

To: We measured the knee extension velocity, quadriceps strength, passive range of motion (ROM), and pain to evaluate knee function. (Lines 115-116)

1. **Please add the units for all your parameters used in your manuscript.**

Response: As the reviewer suggested, we have added the units for all parameters in the Measurements section as follows.

- Gait speed was expressed in meters per second (m/s). (Lines 122-123)
- The time required to complete TUG was measured in seconds (sec) using a stopwatch. (Lines 128-129)
- We measured the angular velocity of knee extension in degrees per second (°/s). (Lines 141-142)
- The patient was instructed to extend the knee with as much force as possible for 3 s, and the strength was measured in kilogram (kg). (Lines 152-153)
- Passive knee flexion and extension ROMs were measured using a goniometer in degrees (°) with the patient in the supine position and recorded to the nearest 5°. (Lines 161-162)

1. **Please add reference to justify the nearest of 5° using the goniometer.**

Response: Thank you for your suggestion. We have added the reference.

To: Passive knee flexion and extension ROMs were measured using a goniometer in degrees (°) with the patient in the supine position and recorded to the nearest 5° [29]. (Lines 161-162)

29. Roach KE, Miles TP. Normal hip and knee active range of motion: the relationship to age. Phys Ther. 1991;71(9): 656-665. doi: 10.1093/ptj/71.9.656.

**Statistical section:**

1. **Please add that you also used percentages of the description of your cohort. Define also SD.**

Response: Thank you for emphasizing this. I have added a description about using percentage and the definition of SD.

From: Descriptive statistics were calculated for all variables and reported as the mean ± SD.

To: Descriptive statistics were calculated for all variables and all continuous variables are expressed as means and standard deviations (SD). Only sex was shown in percentages. (Lines 169-170)

1. **Concerning the correlations, please add a reference to explain the strength of the link between the data.**

Response: In accordance with the reviewer's comment, we have added an explanation about the strength of correlation coefficient and a reference.

To: Pearson correlation coefficients were classified using the following definitions: 0–0.19, very weak; 0.2–0.39, weak; 0.40–0.59, moderate; 0.60–0.79, strong; and 0.80–1.0, very strong [30]. (Lines 173-175)

30. Frankland MP, Dillman JR, Anton CG, Coley BD, Nasser MP, O'Hara SM, et al. Diagnostic performance of ultrasound hepatorenal index for the diagnosis of hepatic steatosis in children. Pediatr Radiol. 2022;1-8. doi: 10.1007/s00247-022-05313-x.
2. **Did you test uni-regression before to do your model? How you justify the choice of your co-variate in your model? Did you test the correlation between each co-variates before your final model?**

Response: We thank the reviewer for this insightful comment. This study aimed to identify the importance of the knee extension velocity in determining the gait function after TKA. Therefore, we need to enter bilateral quadriceps strength and pain as covariates in the same model, along with knee extension velocity. As the reviewer pointed out, it is necessary to check for multicollinearity; hence, we added to the Statistical analyses section and Table 4.

To: We calculated the variance inflation factor (VIF) to evaluate multicollinearity and multicollinearity may be present when the VIF is > 5 [31]. (Lines 178-180)

31. Cohen J, Cohen P, West SG, Aiken LS. Applied Multiple Regression/Correlation Analysis for the Behavioral Sciences. 2nd ed. Oxfordshire: Taylor & Francis; 2013.

***Results**

1. **Regarding the range of your BMI, you have a large range and very little SD, did you cheeked your data to explain this important difference?**

Response: Thank you for pointing this out. We checked our data again and there were no errors. We provide the histogram of BMI.

1. **Table 3, please precise in your results that the correlation are indeed significant but always moderate or low … it is important to indicate this information, to always think in terms of clinical approach.**

Response: We appreciate the reviewer’s comment. We have added a description about the strength of the correlation coefficient in the Results section.

From: Both bilateral knee extension velocities and bilateral muscle strengths were significantly correlated with gait speed and TUG at all time points.

To: Both bilateral knee extension velocities and bilateral muscle strengths showed weak-to-moderate correlations with gait speed and TUG at all time points. (Lines 203-205)

***Discussion
This section is well written.**

1. **Please modify this sentence because we think that the knee extension velocities have been recorded directly during the gait with reflective markers for examples : “In the present study, the maximum knee extension velocities on the operated side were 340.9 °/s at 1.01 m/s in gait speed (2 weeks) and 373.7 °/s at 1.12 m/s (3 weeks).“**

Response: As the reviewer noted, our original expression was confusing. To avoid confusion, we have shown the results for the knee extension velocity and gait speed separately, and knee extension velocity during gait and sitting was clearly described.

From: In the present study, the maximum knee extension velocities on the operated side were 340.9 °/s at 1.01 m/s in gait speed (2 weeks) and 373.7 °/s at 1.12 m/s (3 weeks). These results indicate the knee extension velocity of the present study is close to the knee extension angular velocity during gait.

To: In the present study, gait speed was 1.01 m/s at 2 weeks and 1.12 m/s at 3weeks after TKA and the knee extension velocities on the operated side in a seated position were 340.9 °/s at 2 weeks and 373.7 °/s at 3 weeks. These results indicate that the knee extension velocity in the sitting position of TKA patients whose gait speeds have recovered to 1.0–1.1 m/s is close to the knee extension angular velocity of healthy people during gait at a speed of 1.0–1.2 m/s. (Lines 243-248)

1. **In the sentence below, I think that it is important to remain cautious in the statements made. This may indeed be an important factor, but the angular velocity during operation depends on many other factors.**

**« This closeness may indicate that the knee extension velocity is a limiting factor of gait speed. This factor may be one reason for the strong relationship between knee extension velocity and gait speed.“**

Response: We agree with the reviewer's comment that we should express our thoughts more cautiously. We have changed this part as follows.

From: This closeness may indicate that the knee extension velocity is a limiting factor of gait speed. This factor may be one reason for the strong relationship between knee extension velocity and gait speed.

To: This closeness may partly explain why the knee extension velocity measured in the sitting position moderately correlated with gait speed. (Lines 248-249)

1. **Concerning the stiff knee, there are several mechanics that can cause this inefficient gait. The first is mainly caused by inappropriate swing-phase activity of RF (necessary to have EMG) referred as spasticity (stroke, CP patients…). The second mechanism may be the abnormal foot kinematic at toe-off with a lack of push-off power at the ankle joint with a consequent lack of passive knee flexion. The third mechanism may be the insufficient hip pull-off that should produce hip flexion during swing…. Finally, there is an important problem of compensation gait for this population of patient with OA during several years…. And also an important impact of the pain or fear of pain with therefore a slow gait and compensation…**

**Maybe it is important to precise this in your manuscript.**

Response: Thank you very much for this insightful comment. We recognize and agree with this comment that the stiff knee is caused by inappropriate activity in RF, lack of push-off, and insufficient hip pull-off. Furthermore, we have been made aware by the reviewer's comment that the pain or fear of pain can be a common limiting factor for gait speed and knee extension velocity. Therefore, we have added a sentence about pain or/and fear of pain.

From: These indicate that quadriceps/hamstrings co-activation is a common determinant of gait speed and knee extension velocity. Therefore, we consider this commonality an underlying mechanism of the significant relationship between gait function and knee extension velocity following TKA.

To: These indicate that quadriceps/hamstrings co-activation is a common determinant of gait speed and knee extension velocity. In addition, pain or/and fear of pain can also be common limitations of gait speed and knee extension velocity. We consider these commonalities an underlying mechanism of the significant relationship between gait function and knee extension velocity following TKA. (Lines 259-263)

We wish to thank the Reviewer again for his/her valuable comments.

**Responses to Reviewer #2:**

We thank you for the time and effort spent in reviewing our manuscript and suggesting some important points to consider. **(Excerpts from the reviewer’s comments are indicated in bold.)**

First of all, the representation of the knee angle was confusing because we used mixed expressions for the knee flexion angle and knee joint angle. In the revised submission, we have unified the use of knee joint angles. Additionally, we stated the endpoint for measuring knee extension velocity as 170° of knee extension; however, this was an error of 160° in a knee joint angle. We revised our manuscript as follows.

From: We made a target by wrapping a soft cushion on the frame, and it was set such that the center of the lower leg should hit it at 170° of knee extension. The patient was then instructed to extend the knee joint as quickly as possible from 90° of flexion to hit the target without any external load.

To: We made a target by wrapping a soft cushion on the frame, and it was set such that the center of the lower leg should hit it 160° of the knee joint angle. The patient was then instructed to extend the knee joint as quickly as possible from 90° of the knee joint angle to hit the target without any external load. (Lines 138-141)

**Reviewer #2: I have strong reservation on the methods. Not much details of the measurement and data processing techniques are provided in this paper.**

**1. The authors rightly pointed out that muscle power (a produce of strength and velocity) is important in clinical rehabilitation. I wonder why the authors did not examine power but single out the velocity component. Can they compare power, strength and velocity? Rather than advocating one better than another, will the variables collectively inform the clinicians better?**

Response:

As noted by the reviewer, we designed this study to confirm the importance of the velocity component to gait function in the early postoperative period following TKA. The reason for setting this objective is that the standard rehabilitation program focuses on the strength component but not the velocity component. Since the importance of power and strength components to gait function has been compared in previous studies (Bean JF, 2003), we thought it would be possible to compare the velocity component to muscle strength.

As the reviewer pointed out, it is certainly not easy to separate the muscular and velocity component completely. Therefore, to separate each component as possible, strength was measured with the velocity set to 0 (isometric contraction), and velocity was measured with the external load set to 0. Consequently, the importance of the velocity component to gait function was shown in this study. We believe that considering the velocity component in rehabilitation programs may be useful in restoring gait function in the early postoperative period following TKA.

**2. Handhold dynamometer is not the best for knee extension testing as it is hard to stabilize the joint. Did the authors check the joint angles during the tests using objective methods? It is likely that the leg will move away from the initial start position of 70 degrees of knee flexion.**

Response:

We used a hand-held dynamometer (HDD) equipped with a fixing belt (μ-Tas F-1, Anima Corp., Tokyo, Japan). We show two figures from previous studies using this HDD (Katoh M, 2009 and Kiyohara M). Using a belt improved the reliability of isometric strength measurement using HDD, which has proven to be sufficiently reliable (Katoh M, 2009). In this method, the joint angle can be set by adjusting the length of the belt. In the present study, we have checked the knee joint angle and adjusted the length of the belt to set 110° in one practical trial.

As pointed out by the reviewer, there was a lack of explanation regarding the measurement of isometric muscle force. In addition, the text “The patient was seated with their knees positioned at 70° flexion.” was also misleading. Therefore, we have added the following explanation.

(Katoh M. et al, J phys Ther Sci. 2009)

(Kiyohara M, Eur J Orthop Surg Traumatol. 2019)

From:

Quadriceps muscle strength was defined as voluntary isometric knee extension force. We measured the strength on both the operated and non-operated limbs as follows. The patient was seated with their knees positioned at 70° flexion. The patient was instructed to extend the knee with as much force as possible for 3 seconds, and the strength was measured using a hand-held dynamometer (μTas F-1, Anima, Tokyo). This test was performed twice after one practice trial, and the larger value was used for the subsequent analyses.

To:

Quadriceps muscle strength was defined as a voluntary isometric knee extension force. We measured the strength of both the operated and non-operated limbs. Figure 2 shows the setup for measuring the quadriceps strength. We used a hand-held dynamometer (HDD) with a fixing belt (μ-Tas F-1, Anima Corp., Tokyo, Japan). The patient was seated upright with their arms crossed. The sensor was set in the dynamometer in front of the distal leg with a belt. The patient was instructed to extend the knee with as much force as possible for 3 s, and the strength was measured in kilogram (kg). The knee joint angle was set at 110° during the measurement. To match the knee joint angle to 110°, the measurer adjusted the length of the belt during a practice trial. This test was performed twice after one practice trial, and the larger value was used for the subsequent analyses. (Lines 147-157)

**3. What is the reliability of the equipment and testers in this study?**

Response: We thank the reviewer for this comment. We have preliminarily verified the inter- and the intra-rater reliability of knee extension velocity and quadriceps muscle strength in young people (n=11). The intraclass correlation coefficient (ICC) was used to evaluate the reliability. As a result, the reliability showed a high-class correlation coefficient in almost all values as follows. Therefore, we used both tests in this study.

**4. I was under the impression that the knee extension strength was measured as isometric contraction (knee flexion angle fixed at 70 degrees as much as possible). How did the authors measure velocity for isometric extension using a handhold dynamometer? This part is not clear.**

Response: We appreciate the reviewer's comment on this point. We separately measured "knee extension velocity" and "quadriceps isometric strength" in this study. We used a gyro-sensor to measure the velocity when the patient extended their knee as fast as possible from a knee joint angle of 90° to 160° and a hand-held dynamometer to measure the strength at knee joint angle of 110°. To make this point clearer, we have revised Figure 1 (velocity) and added Figure 2 (strength).

**5. Can a picture be provided to illustrate the test protocol? There was a figure at the end but rather strange. The angle there definitely**

Response: In accordance with the reviewer's comment, we have changed Figure 1 and added Figure 2.

Fig 1. Experimental setup for measuring the maximum knee extension velocity.

Fig.2 Experimental setup for measuring the quadriceps muscle strength.

**6. Please prove more details on how raw data are processed and how each variables (e.g. velocity) are calculated or extracted.**

Response:

We measured knee extension velocity using a wireless gyro-sensor. The sampling rate of the gyro-sensor was 200 Hz and the measurement data were transferred to a personal computer. As suggested, we have added these detailed information to the knee function section.

From: The subject was seated on an elevated bed with a gyro-sensor (MVP-RF8-HC, W45×D45×H18.5 mm, 60 g, MicroStone, Nagano, Japan) on the distal lower limb.

To: The subject was seated on an elevated bed with a wireless gyro-sensor (MVP-RF8-HC, W45×D45×H18.5 mm, 60 g, MicroStone, Nagano, Japan) on the distal lower limb. The sampling rate of the gyro-sensor was 200 Hz and the measurement data were transferred to a personal computer. (Lines 134-138)

**7. I am unsure about the data in Table 2. Knee extension velocity of over 400 degrees per second – during what movement? How?**

Response:

We measured the knee extension angular velocity using a gyro-sensor when patients with TKA extended their knee as fast as possible from a knee joint angle of 90° to 160° in the sitting position. The knee extension velocity was measured in 113 healthy elderly subjects with an average age of 72.5 years (599.2 ± 108.4°/s [unpublished]). Considering the age and the velocity values, we believe that the data from the TKA patients in the present study are accurate.

**8. I did not read further than the results because I have some doubt about the methodology and data integrity.**

Response:

Again, we deeply appreciate the reviewer for her/his time on our study and we apologize for the confusion. As mentioned above, we exerted all efforts to clarify the methods in detail. Therefore, now, we deem that it is much clearer for the readers.
