# Supplementary material for: Maximum knee extension velocity without external load is a stronger determinant of gait function than quadriceps strength in the early postoperative period following total knee arthroplasty
Source: PLoS One. 2022 Nov 22;17(11):e0276219. doi: 10.1371/journal.pone.0276219 (PMC9681062; doi:10.1371/journal.pone.0276219)
Supplement: Attachment — Submitted filename: Response to Reviewers.docx [file pone.0276219.s003.docx]

**Reviewer #2: The authors put in a lot of effort to clarify my previous concerns. They also added more figures and text to better explain their procedures in the manuscript.**

Response: Thank you for acknowledging our effort. We would like to express our appreciation to the reviewer for providing these constructive suggestions that have helped us to greatly improve our manuscript. We have revised the manuscript in accordance with the reviewer’s suggestions.

**1. Did the authors use tolerance value in addition to VIF? What do they mean by “Multicollinearity may be present”? The word “maybe” is rather vague – do the authors use a cut-off value of VIF 5 to make some decision? Please be very clear on the interpretation and steps to take when VIF is greater than 5.**

Response: We thank the reviewer for the insightful comment. We used the VIF to check for multicollinearity among the independent variables, with a VIF>5 indicative of multicollinearity (Cohen J, 2013). If multicollinearity was observed, we assumed that the variables should be removed from the model. However, as none of the variables in any model used in this study showed a VIF of more than five, no variable was removed. We agree with the reviewer that our expression regarding this issue may be unclear, so we have changed it. Furthermore, as we did not mention VIF in the Results section, we have added a brief description.

The changes are as follows:

**(From)**

Methods: We calculated the variance inflation factor (VIF) to evaluate multicollinearity and multicollinearity may be present when the VIF is > 5 [31].

**(To)**

- Methods section (Lines 185-187):

We calculated the variance inflation factor (VIF) to check for multicollinearity, and any independent variables for which VIF was greater than 5 were removed from the model [31].

- Results section (Lines 217-218):

As all VIF values were lower than 5, we confirmed the absence of multicollinearity in both models.

**2. Table 1 – This table can be expanded to include data of males only, females only and all participants as a group. It will be good if other demographic characteristics (e.g. how many use a walking stick, medical history, time from operation) can be included if such data are available.**

Response: As suggested, we have added sex-specific data for males and females only to Table 1. Unfortunately, data regarding a walking stick, medical history, and time since surgery, were not available. We aim to collect this data in future studies.

| **Table 1. Preoperative characteristics of the patients** | | | | | | | | | | | | | | | | | | | | | | | |
| --- | --- | --- | --- | --- | --- | --- | --- | --- | --- | --- | --- | --- | --- | --- | --- | --- | --- | --- | --- | --- | --- | --- | --- |
|  | Male (n = 43) | | | | | | |  | Female (n = 143) | | | | | | |  | All (n = 186) | | | | | | |
|  | Value | | |  | Range | | |  | Value | | |  | Range | | |  | Value | | |  | Range | | |
| Age (years) | 75.8 | ± | 7.4 |  | 62 | – | 92 |  | 75.9 | ± | 6.4 |  | 60 | – | 90 |  | 75.9 | ± | 6.6 |  | 60 | – | 92 |
| Height (cm) | 161.1 | ± | 7.8 |  | 148 | – | 176 |  | 148.2 | ± | 5.7 |  | 136 | – | 161 |  | 151.2 | ± | 8.3 |  | 136 | – | 176 |
| Weight (kg) | 67.0 | ± | 10.7 |  | 46.0 | – | 90.2 |  | 58.0 | ± | 8.9 |  | 36.7 | – | 83.5 |  | 60.1 | ± | 10.0 |  | 36.7 | – | 90.2 |
| BMI (kg/m^2^) | 25.8 | ± | 3.8 |  | 18.1 | – | 34.8 |  | 26.5 | ± | 4.0 |  | 17.3 | – | 47.8 |  | 26.3 | ± | 3.9 |  | 17.3 | – | 47.8 |
| Mean ± SD, BMI: Body Mass Index | | | | | |  |  |  |  |  |  |  |  |  |  |  |  |  |  |  |  |  |  |

**3. Line 248 – Please rephrase “This closeness”.**

Response: As suggested by the reviewer, we have changed from “This closeness” to “The proximity of the two values”.

- Discussion Section (Line 256-258):

The proximity of the two values may partly explain why knee extension velocity measured in the sitting position was moderately correlated with gait speed.

**4. In the Discussion, the authors wrote one paragraph on “Secondly, we focused on the common mechanism to determine the knee extension velocity and the knee motion during gait….” However, after reading the entire paragraph which is primarily a summary of other studies, it is puzzling what the present study contributed to the mechanism. It would be better if the authors can relate the findings from their own study with the literature in a more explicit manner.**

Response: Thank you for your insightful comment. As suggested, to relate our findings with those of previous studies, we have edited and reconstructed the Discussion section and added more detailed explanations.

- Discussion Section (Line 259-277):

Secondly, we focused on the common mechanism to determine knee motion during gait and knee extension movement during the velocity measurement. The kinematic characteristics of gait in patients with TKA have reduced knee flexion during the loading response, reduced knee extension during the late stance phase, and decreased knee flexion during the swing phase [34,35]. In other words, the range of knee motion during gait is smaller than that in healthy elderly adults, and it has been called "stiff knee gait" [36-38]. Stiff knee gait leads to shortening of the step length and ultimately slower gait speed [8]. One of the main etiologies of stiff knee gait is quadriceps/hamstrings co-activation [36,37], and thus quadriceps/hamstrings co-activation can be assumed to have an effect on gait speed. Conversely, increased muscle co-activation also contributes to decreased maximum movement velocity in a single joint [39], so quadriceps/hamstrings co-activation may be a limiting factor for knee extension velocity during measurement in patients with TKA. From the above, quadriceps/hamstrings co-activation is considered a common determinant of both knee motion during gait and knee extension velocity during measurement. Furthermore, pain or/and fear of pain can also be common limitations of gait speed and knee extension velocity. We consider these commonalities an underlying mechanism of the significant relationship between gait function and knee extension velocity following TKA. Furthermore, it could be said that a decrease in knee extension velocity is directly related to decrease in gait speed.

**5. The authors demonstrated experimentally that knee extension velocity on the operated side is a better predictor of gait function than quadriceps strength. This is nice from a research perspective, but is there any practical implication? In clinical practice, it is a lot easier to measure gait speed and TUG than knee extension velocity or even quadriceps strength. Thus, it is unlikely that clinician will measure knee extension velocity. Are there any direct clinical implications from this study?**

Response: We understand the reviewer’s concern that measuring knee extension velocity using a gyro-sensor is more complicated than measuring gait speed, TUG, or even quadriceps strength. However, a recent study showed that angular velocity could be easily measured with a smartphone application, although there is limited validation to support its accuracy (Hughes GTG, 2021). Therefore, we believe that the measurement of knee extension velocity will become more accessible in the future, allowing it to be performed in clinical situations.

Quadriceps strength is considered a primary determinant of gait function, particularly early after TKA. However, as noted in the Introduction, there is often a large gap between quadriceps weakness and the recovery of gait function. Our results revealed that knee extension velocity on the operative side is a more critical determinant of gait function than quadriceps strength, indicating that rehabilitation focusing on knee velocity may be more effective. Therefore, we plan a future study focusing on knee extension velocity. If significant improvements in gait function are achieved, we believe that there will be a paradigm shift in clinical rehabilitation after TKA.

**6. In response to the reviewers’ previous comments, the authors presented a figure on the velocity raw data from the gyro-sensor. It will be nice to incorporate this figure together with Figure 1 to comprehensively show to test procedures (start/end position, sensor, data).**

**Response:** As the reviewer suggested, we have summarized the setup for the measurement, sensor, and representative data in Figure 1.

**Fig 1. Measurement of the maximum knee extension velocity**

(A, B) Experimental setup for measurement. The patient was seated on an elevated bed with a wireless gyro-sensor, where they extended their knee joint five times as quickly as possible from 90°(A) to 160°(B) to hit the soft cushion. (C) The gyro-sensor. (D) Representative data of knee extension velocity. The red arrow indicates the maximum knee extension velocity of five times. (Lines 147-152)
